# Supplementary material for: An epigenetic clock for Xenopus tropicalis
Source: NPJ Aging. 2025 May 22;11(1):38. doi: 10.1038/s41514-025-00236-x (PMC12098715; doi:10.1038/s41514-025-00236-x)
Supplement: Supplementary file 1 — Supplementary information [file 41514_2025_236_MOESM1_ESM.pdf]

Supplementary information

Supplementary Data 1: *Xenopus* samples description
Supplementary Data 2: Targeted bisulfite sequencing probes
Supplementary Data 3: Genetic variation
Supplementary Data 4: Epigenetic clock weights
Supplementary Data 5: Fisher's exact test results
Supplementary Data 6: Wilcoxon rank-sum test results

Supplementary Figures

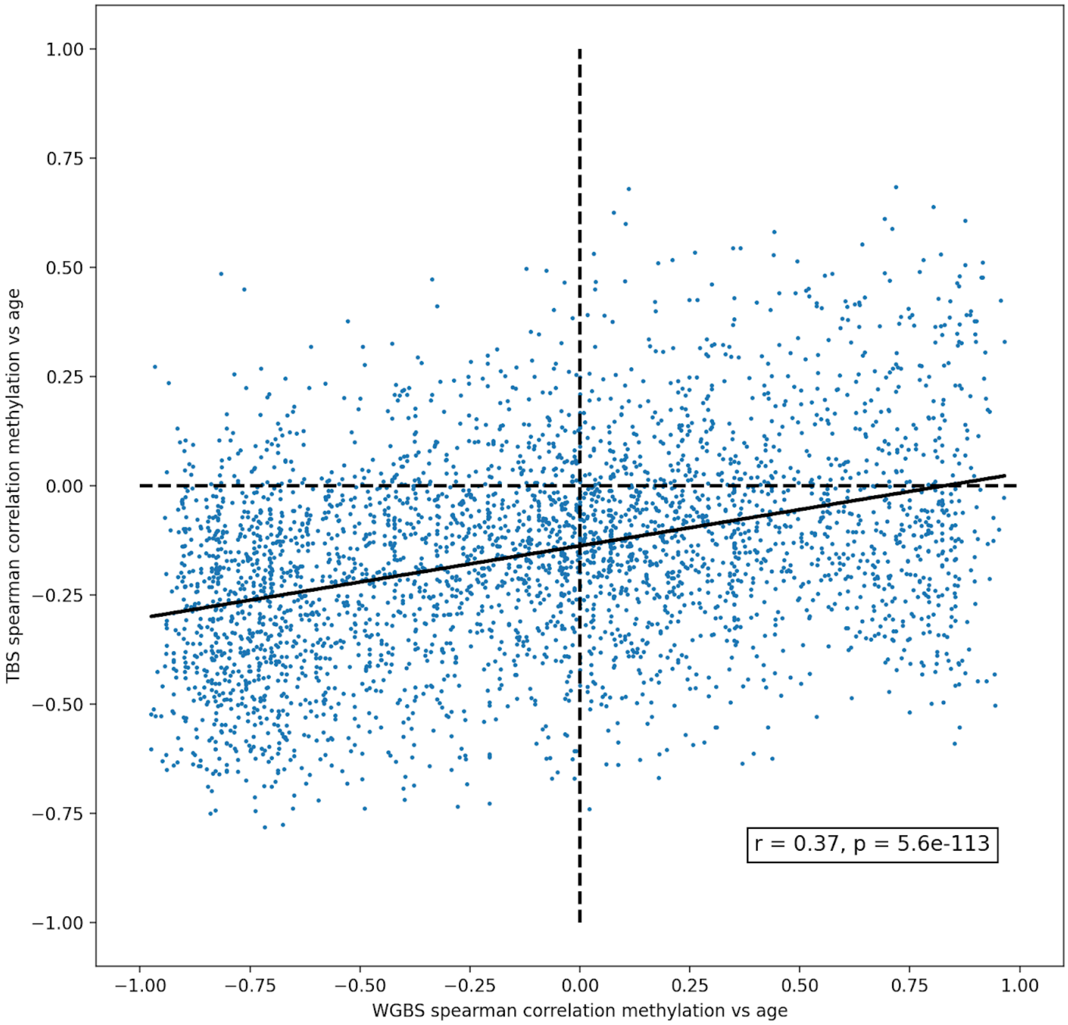

Figure S1: Comparison of n=3506 CpG sites shared between the previously collected whole genome bisulfite sequencing (WGBS) dataset and this study's targeted bisulfite sequencing (TBS) dataset. The Spearman correlation between methylation and age for the corresponding dataset is shown on each axis. The Pearson correlation between the Spearman correlations (WGBS vs TBS) in the plot is r=0.37 ($p=5.6\times10^{-113}$, n=3506). The solid black line shows the least squares regression best-fit line.

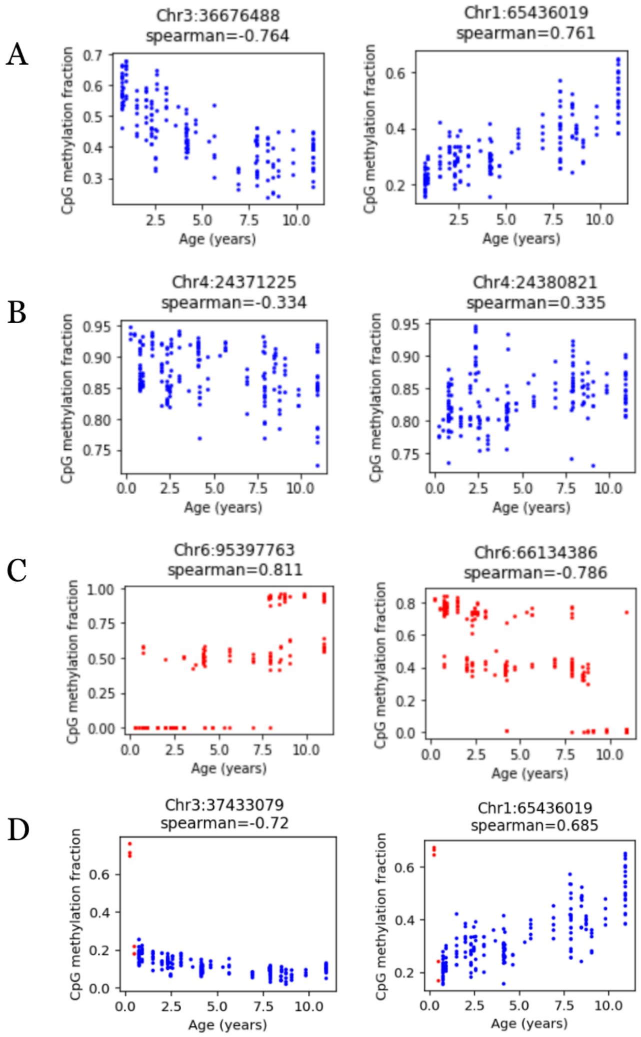

Figure S2: (A) Examples of CpG sites with highly age-correlated methylation (Spearman $r_s \approx \pm 0.75$). **(B)** Examples of CpG sites with lower correlation between methylation and age (Spearman $r_s \approx \pm 0.33$). **(C)** Examples of CpG sites with a pattern of 3 distinct methylation levels. These are filtered out of downstream analyses. **(D)** Examples of CpG sites with abnormally high methylation levels in tadpoles (red). The tadpole samples are removed for some downstream analyses.

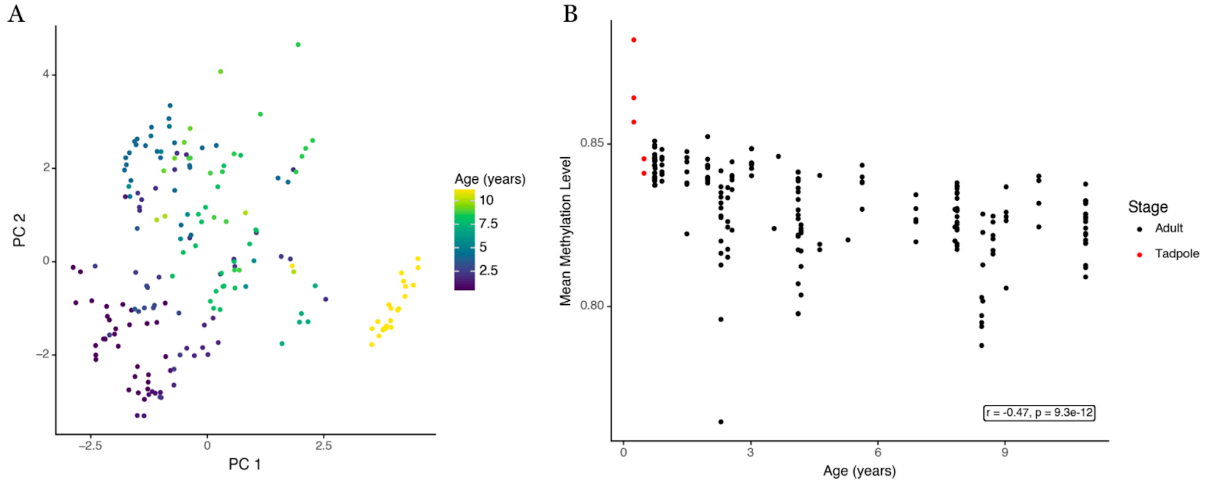

Figure S3: (**A**) Principal component analysis (PCA) of the DNA methylation values for each frog. Each CpG site's methylation value was centered to the mean across all frogs before PCA. (**B**) Mean methylation level across all targeted bisulfite sequencing CpG sites vs age. CpG sites with a minimum coverage of 100 were used for both plots.

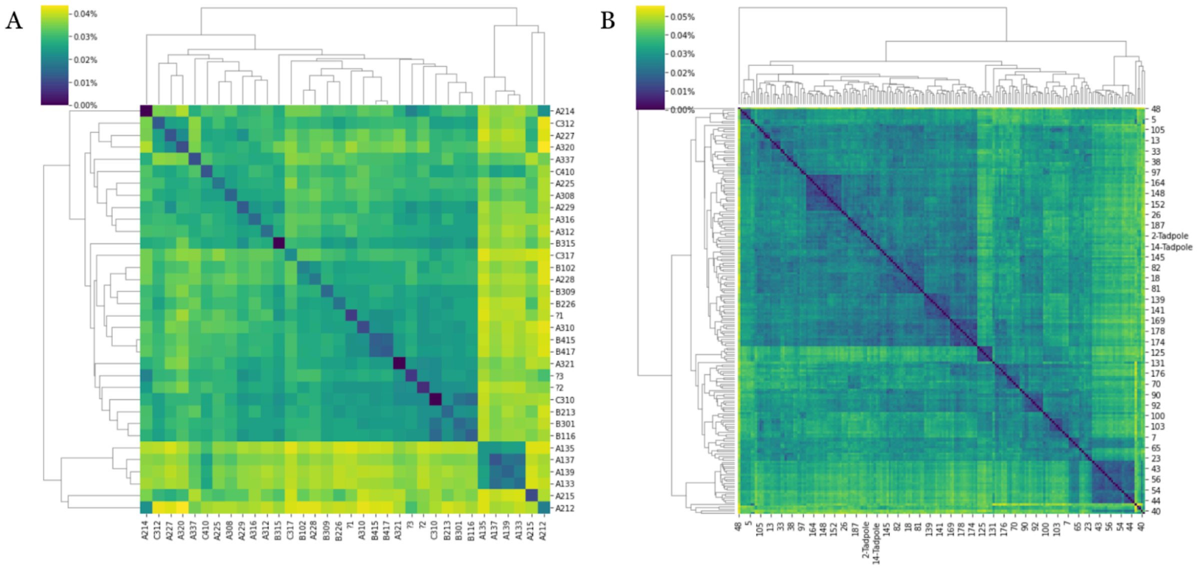

Figure S4: (**A**) Genetic variation between every pair of tanks (average number of SNP differences / number of callable base pairs). (**B**) Genetic variation between every pair of frogs (number of SNP differences / number of callable base pairs).

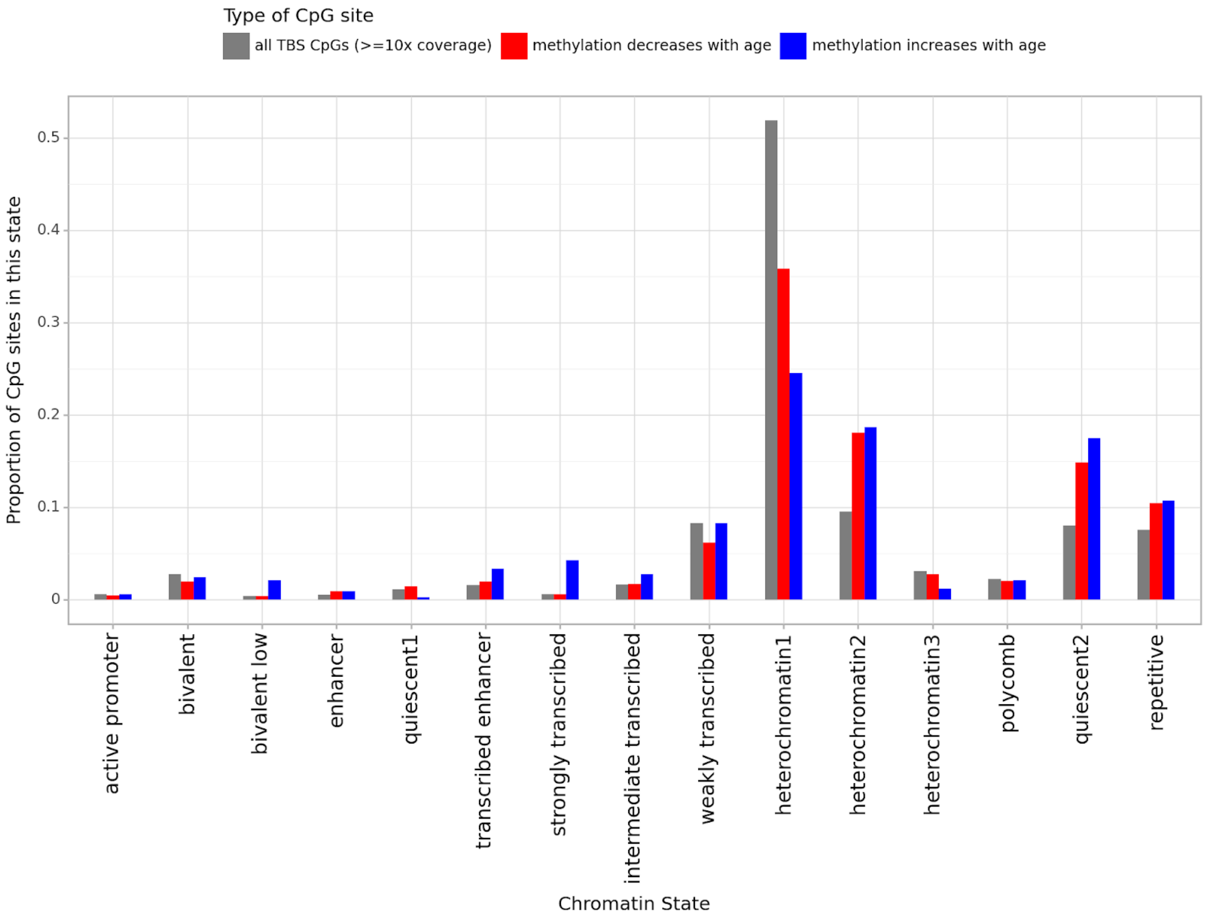

Figure S5: Proportion of CpG sites in each chromatin state for 3 groups of CpGs. There are 25,362 CpGs in the TBS dataset with >= 10x coverage (gray), 1,907 highly negatively age-associated CpGs (red), and 326 highly positively age-associated CpGs (blue). 3
